# Correction: Antischistosomal, antionchocercal and antitrypanosomal potentials of some Ghanaian traditional medicines and their constituents

**DOI:** 10.1371/journal.pntd.0011044

**Published:** 2023-01-04

**Authors:** Emmanuella Bema Twumasi, Pearl Ihuoma Akazue, Kwaku Kyeremeh, Theresa Manful Gwira, Jennifer Keiser, Fidelis Cho-Ngwa, Adrian Flint, Barbara Anibea, Emmanuel Yeboah Bonsu, Richard K. Amewu, Linda Eva Amoah, Regina Appiah-Opong, Dorcas Osei-Safo

There is an error in affiliation for authors Pearl Ihuoma Akazue and Theresa Manful Gwira. The correct affiliation is: West African Centre for Cell Biology and Infectious Pathogens, Department of Biochemistry, Cell and Molecular Biology, University of Ghana, Accra, Ghana.
