# Supplementary figures and images for: Antischistosomal, antionchocercal and antitrypanosomal potentials of some Ghanaian traditional medicines and their constituents
Source: PLoS Negl Trop Dis. 2020 Dec 31;14(12):e0008919. doi: 10.1371/journal.pntd.0008919 (PMC7810346; doi:10.1371/journal.pntd.0008919)

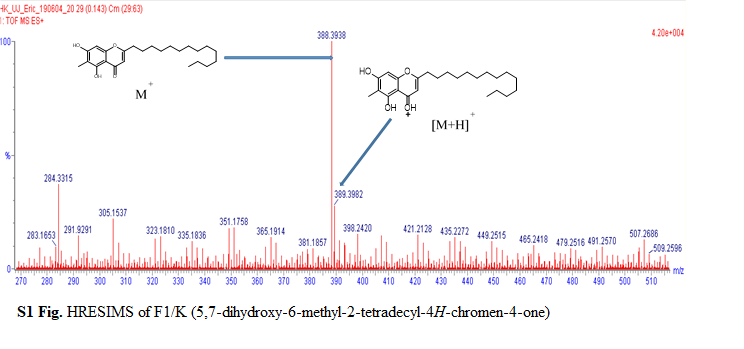

Supplement: S1 Fig — (TIF) [file pntd.0008919.s001.tif]

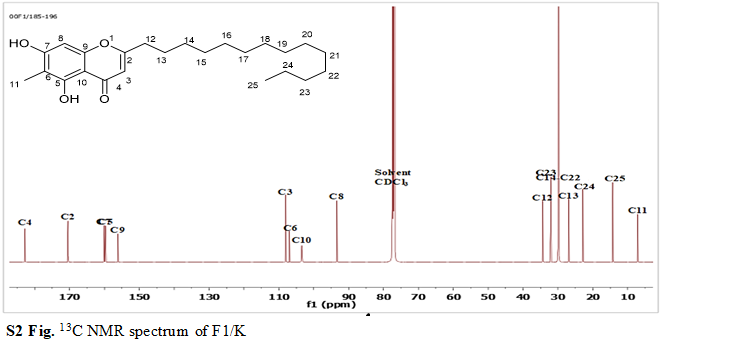

Supplement: S2 Fig — (TIF) [file pntd.0008919.s002.tif]

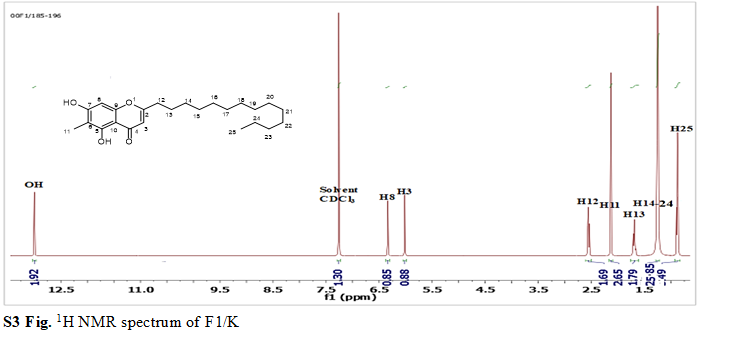

Supplement: S3 Fig — (TIF) [file pntd.0008919.s003.tif]

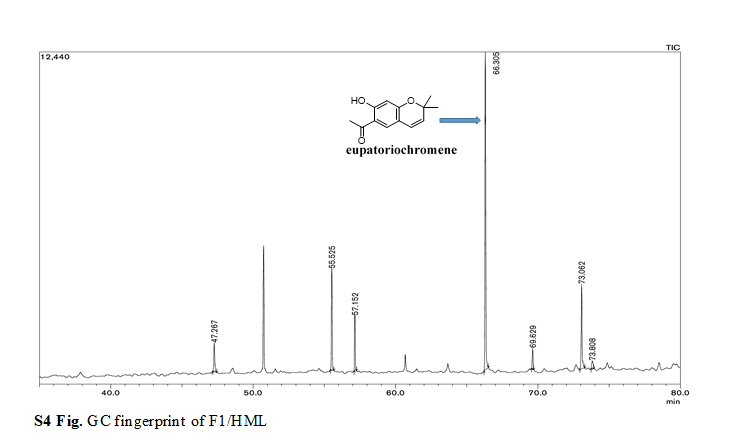

Supplement: S4 Fig — (TIF) [file pntd.0008919.s004.tif]

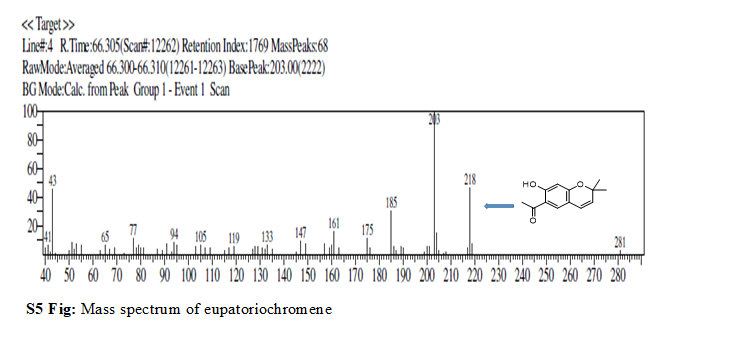

Supplement: S5 Fig — (TIF) [file pntd.0008919.s005.tif]
