# Supplementary material for: Antischistosomal, antionchocercal and antitrypanosomal potentials of some Ghanaian traditional medicines and their constituents
Source: PLoS Negl Trop Dis. 2020 Dec 31;14(12):e0008919. doi: 10.1371/journal.pntd.0008919 (PMC7810346; doi:10.1371/journal.pntd.0008919)
Supplement: Attachment — Submitted filename: To Editors.docx [file pntd.0008919.s006.docx]

I hope that the results described in this work can be published. But describing tests on herbal remedies without naming the plant sources is unacceptable. All other papers on herbal remedies that I have seen give the species. Perhaps Bill Gates could bribe the traditional practioners to release information?

Please note that I am not a chemist and cannot judge the accuracy of the proposed structure. Also that my list of queries is far from complete.
