# Supplementary material for: Antischistosomal, antionchocercal and antitrypanosomal potentials of some Ghanaian traditional medicines and their constituents
Source: PLoS Negl Trop Dis. 2020 Dec 31;14(12):e0008919. doi: 10.1371/journal.pntd.0008919 (PMC7810346; doi:10.1371/journal.pntd.0008919)
Supplement: Attachment — Submitted filename: Comments to the Authors.docx [file pntd.0008919.s007.docx]

1. Using the standard approach of fractionating extracts of traditional medicines and testing them in vitro on pathogens, the authors have identified a compound that is more effective than the standard drug, diminazene, against trypanosomes. This is a considerable achievement, and a promising contribution to the development of new drugs.

2. The work compares the efficacies of extracts of 15 traditional medicines against *Schistosoma mansoni*, *Onchocerca ochengi* and *Trypanosoma brucei brucei*. This information might have been useful, but is not, because the biological sources (species and part of plant) from which the active compounds were extracted are not given. Although traditional medicine practitioners may consider that they gain prestige by possessing trade secrets, the hurt to the public good is enormous.

2.1. Other articles (apparently *all* other articles) on traditional plant-based medicines from Ghana give the species (e.g. Baodu & Akase 2017 ref.25; Soelberg et al 2015 J Ethnopharmacology 160:109; Ragosta et al. Ethnobiology Letters 6(1) 66.)

2.2. Other labs cannot repeat and develop this work (apart from the identified compound).

2.3. Traditional medicine itself would be improved if all practioners adopted the best practice, and this could only be done if known preparations are compared.

2.4. Secrecy that hinders public health is counter to the ethos of the PLoS journals.

3. P.8. "no biological activity assay against LF was undertaken". To include lymphatic filariasis in the first sentence of the Introduction, but not trypanosomiasis, confuses the reader. Lymphatic filariasis figures in Table 1, so it must be mentioned, but as no more than another important target for which new drugs are needed.

4. Table 1. The authors are to be commended for winning the confidence of practioners of traditional medicine. However, establishing the efficacy of a treatment is notoriously difficult. It requires blinded allocation of patients to test and control groups (for the placebo effect and natural recovery) and blinded judgement of the outcomes. Despite the footnote, the figures in the last column of Table 1 do not begin to meet scientific criteria. By definition, traditional medicines have probably been used for centuries. This fully justifies investigation of their efficacy, but not any assumption that they *are* effective. The last column should be omitted or given a different title.

5. Extraction procedure. Could the authors please explain this more.

5.1.L.1. In what volume of solvent was the powdered herb macerated?

5.2. Does '24 hours x 3' mean soaking in solvent for three days or that the solvent was changed daily for three days?

5.3. L. 4. What filter was used?

5.4. L. 4. "the crude extracts which were then concentrated". But the filtrates had already been dried.

5.5. L. 5. At what temperature were the extracts stored and for how long?

6. In vitro antischistosomal activity screening

6.1. L.2. In what was the solution in DMSO diluted to give a final concetration of 1%?

6.2. L.6. What is 'M199' medium?

antibacterial/antifungicidal mix. The percentages add up to more than 100%

6.3. What is RPMI 1640?

6.4. What was the concentration of DMSO in the drug stock solution?

6.5. P.10, L.1. "The crude extracts were dissolved in 100% DMSO in a supplemented M199 medium" How can you have 100% DMSO in a M199 medium?

6.6. P.10, L.4. "25 µL of 10% (v/v) DMSO in 175 µL of supplemented M199 medium" What was the remaining 90% of the 25 µL, besides DMSO?

6.7. P.10, L.7."72 hours after drug incubation". Was this supposed to say "after 72 h

incubation"?

6.8. P.10. L.7. Was the procedure single-blind or double-blind?

6.9. P.10, L.14. What is the "10mg" of "10 mg/mL". Extract or extract dissolved in DMSO? What solution was this in? Is this stock solution different from that for the newly transformed schistosomulae?

7. P.14. Chemical profiling. It would be useful to have references to more detailed descriptions of the GCMS, NMR and MS

8. P. 21 "Bioactivity-guided fractionation" L.5. "flash chromatography" should at least be mentioned, as such, in the Methods.

9. Table 4. What n was used to calculate the SD?

10. P.22. "Chemical profiling" Define more closely (unless it is defined earlier).

11. P.23. L.4. "was evident from the IR spectrum". This seems to be the first time we have heard of IR spectral analysis.

12. Fig.1. Some readers may not know what the abbreviations 'COSY' (=COrrelated SpectroscopY) and 'HMBC' (= heteronuclear multi-bond correlation) mean

13. S1 Please define HRESIMS (= high resolution electrospray ionisation mass spectroscopy)

14. S4. The text is illegible.

It would be helpful to reviewers if the lines were numbered (consecutively from the beginning).

Jonathan A. Coles
