# Supplementary material for: Antischistosomal, antionchocercal and antitrypanosomal potentials of some Ghanaian traditional medicines and their constituents
Source: PLoS Negl Trop Dis. 2020 Dec 31;14(12):e0008919. doi: 10.1371/journal.pntd.0008919 (PMC7810346; doi:10.1371/journal.pntd.0008919)
Supplement: Attachment — Submitted filename: My Review comments PLOS 1.doc [file pntd.0008919.s008.doc]

**Antischistosomal, antionchocercal and antitrypanosomal potentials of some Ghanaian
traditional medicines and their constituents (PNTD-D-20-00655)**

Onchocerciasis, schistosomiasis and lymphatic filariasis feature among the most prevalent NTDS and are of top priority to elimination programs. The Expanded Special Programme for Elimination of Neglected Tropical Diseases (ESPEN) recently promotes an integrated approach to eliminating Neglected Tropical Diseases and its strategy is optimisation and expansion of existing drugs to hypo-endemic areas, plus potential integration of alternative strategies.

This manuscript by Twumasi et al fits into the goals of ESPEN and addresses key issues that affect Africa. They evaluated the ‘antischistosomal, antionchocercal, and antitrypanosomal potentials of some Ghanaian traditional medicines and their constituents’ of fifteen traditional medicines in vitro. They carried out cytotoxicity assessment of the extracts and bioassay-guided fractionation of the most active antitrypanosomal extract which yielded an oil (eupatoriochromene).

The following points should be considered to enhance the quality and completeness of the manuscript

**Specific points**

1) Table 1, number of people successfully treated is not an appropriate heading since you said earlier that ‘According to the practitioners, their efficacy claims are based on the number of people who experience reduction or relief from symptoms as a result of using their products’. So it should be coined as the number of people relieved of symptoms and not successfully treated. Given that no laboratory tests were done to determine parasite loads, it is subjective to conclude that the patients were treated successfully
2) Cytotoxicity assessment of extracts. The 50% cytotoxic concentrations (CC50) of the cells were determined by microscopic examinations.

Looking at Table 4, the cytotoxicity was done at 200 µg/mL only. So the CC50 was not determined as stated in the methodology. The selectivity index (SI) column should be included if the CC50 was determined. Also, consider including this aspect in your abstract

3) Statistical analysis for comparisons of different extracts and concentrations for onchocerca and schistosoma will enhance the credibility of findings given that you ran the assays in quadruplicates and triplicates repeatedly.This will reveal if the differences observed are statistically significant in different concentrations or at the IC50. A good example is Table 5.
4) Looking at the results you obtained for the antionchocercal activity of extracts, all the active extracts are dichloromethane extracts. Most filaricidal activities of plants tested come from non-polar extracts. This could be backed up in the discussion section with publications from other authors who had similar findings.

**Minor Points**

5) We recommend that the quality of traditional medicines be improved (Author summary)
6) Primary screening of adult worms and microfilariae, Secondary screening on adult worms

It should be “screening against adult worms” instead of “screen of adult worms”

7) Correct from GCMSNMR to GC/MS/NMR in the manuscript as they are different techniques.
8) Discussion section, ‘however, is on the rise and possess’ it should be poses
9) Introduction section ‘high cost of bringing new drugs to the market’
