# Supplementary material for: Antischistosomal, antionchocercal and antitrypanosomal potentials of some Ghanaian traditional medicines and their constituents
Source: PLoS Negl Trop Dis. 2020 Dec 31;14(12):e0008919. doi: 10.1371/journal.pntd.0008919 (PMC7810346; doi:10.1371/journal.pntd.0008919)
Supplement: Attachment — Submitted filename: Responses to Reviewer comments_PNTD-D-20-00655.doc [file pntd.0008919.s009.doc]

**Antischistosomal, antionchocercal and antitrypanosomal potentials of some Ghanaian
traditional medicines and their constituents (PNTD-D-20-00655)**

Dear Editors,

Kindly find below (highlighted in red), itemised responses to the reviewer comments.

**Specific comments from Reviewer #1**

1. The work compares the efficacies of extracts of 15 traditional medicines against *Schistosoma mansoni, Onchocerca ochengi* and *Trypanosoma brucei brucei*. This information might have been useful, but is not, because the biological sources (species and part of plant) from which the active compounds were extracted are not given. Although traditional medicine practitioners may consider that they gain prestige by possessing trade secrets, the hurt to the public good is enormous.

***Response: Following a discussion with the Executives of the Traditional Medicine Practitioners, I have their permission to disclose the identities of the constituent medicinal plants of the remedies analysed. The details are provided in Table 1.***

3. P.8. "no biological activity assay against LF was undertaken". To include lymphatic filariasis in the first sentence of the Introduction, but not trypanosomiasis, confuses the reader. Lymphatic filariasis figures in Table 1, so it must be mentioned, but as no more than another important target for which new drugs are needed.

***Response: The point raised by the reviewer is fair. However, the goal of the study was to collect and test traditional medicines used for the endemic NTDs in Ghana, which include LF. Since LF was a focus of the study, for which purpose anti-LF remedies were collected, it deserved some attention in the earlier part of the introduction. Trypanosomiasis is mentioned later since it not currently endemic but its inclusion in the study is justified.***

***Moreover, the reason for not conducting any biological activity assay against LF has been provided – it was difficult to identify a collaborating lab within the timeframe of the project. I believe that by the time the reader becomes aware that no biological activity assay against LF was undertaken, adequate background would have been provided to highlight the significant disease burden of LF in Ghana, and for which reason it was considered in the study.***

***For these reasons, I request that current format is maintained. On the other hand, if the editors support the omission of details on LF, I will go by that.***

4. Table 1. The authors are to be commended for winning the confidence of practitioners of traditional medicine. However, establishing the efficacy of a treatment is notoriously difficult. It requires blinded allocation of patients to test and control groups (for the placebo effect and natural recovery) and blinded judgement of the outcomes. Despite the footnote, the figures in the last column of Table 1 do not begin to meet scientific criteria. By definition, traditional medicines have probably been used for centuries. This fully justifies investigation of their efficacy, but not any assumption that they are effective. The last column should be omitted or given a different title.

***Response: The column in question has been removed from Table 1.***

5. Extraction procedure. Could the authors please explain this more.

5.1.L.1. In what volume of solvent was the powdered herb macerated?

5.2. Does '24 hours x 3' mean soaking in solvent for three days or that the solvent was changed daily for three days?

5.3. L. 4. What filter was used?

5.4. L. 4. "the crude extracts which were then concentrated". But the filtrates had already been dried.

5.5. L. 5. At what temperature were the extracts stored and for how long?

***Response: The necessary details have been provided under the section on extraction***

***procedure.***

6. In vitro antischistosomal activity screening

6.1. L.2. In what was the solution in DMSO diluted to give a final concentration of 1%?

6.2. L.6. What is 'M199' medium? antibacterial/antifungicidal mix. The percentages add up to more than 100%

***Response: M199 medium is a standard term. Additional information can be obtained at the***

***following website.*** [https://www.thermofisher.com/order/catalog/product/12340030#/12340030](https://www.thermofisher.com/order/catalog/product/12340030#/12340030)

***The mix-up in the percentages have been corrected.***

6.3. What is RPMI 1640?

***Response: RPMI 1640 is also a standard term. Kindly check the following website for more***

***information.***

[https://www.thermofisher.com/order/catalog/product/31800089#/31800089](https://www.thermofisher.com/order/catalog/product/31800089#/31800089)

6.4. What was the concentration of DMSO in the drug stock solution?

6.5. P.10, L.1. "The crude extracts were dissolved in 100% DMSO in a supplemented M199 medium" How can you have 100% DMSO in a M199 medium?

***Response: The issue has been corrected.***

6.6. P.10, L.4. "25 µL of 10% (v/v) DMSO in 175 µL of supplemented M199 medium" What was the remaining 90% of the 25 µL, besides DMSO?

***Response: The details have been provided.***

6.7. P.10, L.7."72 hours after drug incubation". Was this supposed to say "after 72 h

incubation"?

***Response: I believe that there is no difference, so it is a matter of preference where ‘after’***

***is placed.***

6.8. P.10. L.7. Was the procedure single-blind or double-blind?

***Response: No, the procedure was not blinded.***

6.9. P.10, L.14. What is the "10mg" of "10 mg/mL". Extract or extract dissolved in DMSO? What solution was this in? Is this stock solution different from that for the newly transformed schistosomulae?

***Response: It is the extract dissolved in DMSO as indicated under Sample preparation that the crude extracts were dissolved in DMSO.***

***The stock solution is not different from that for the newly transformed schistosomula***.

7. P.14. Chemical profiling. It would be useful to have references to more detailed descriptions of the GCMS, NMR and MS

***Response: For GC/MS, the following details are provided:***

***1) make of the instrument,***

***2) column type and dimensions,***

***3) carrier gas,***

***4) temperature ramp,***

***5) sample preparation,***

***6) identification library***

***7) place of analysis***

***For NMR, the following details are provided:***

***1) make of the instrument,***

***2) frequency of the instrument,***

***3) solvent used,***

***4) internal reference standard***

***5) type of experiments run***

***6) place of analysis***

***For Mass spectrometry, the following details are provided:***

***1) make of the instrument,***

***2) ionization technique,***

***3) voltage used,***

***4) place of analysis***

***If any other specific details are required, I will happy to include them.***

8. P. 21 "Bioactivity-guided fractionation" L.5. "flash chromatography" should at least be mentioned, as such, in the Methods.

***Response: Flash chromatography is appears under ‘Bioactivity-guided fractionation of NTD-B4-DCM’, in line 551 of the document with track changes and line 400 in the document with no track changes.***

***Moreover, the extraction process has been presented with greater detail.***

9. Table 4. What n was used to calculate the SD?

***Response:*** ***Experiments were done in quadruplicates for adult worms and duplicates for microfilariae. SD values have been included.***

10. P.22. "Chemical profiling" Define more closely (unless it is defined earlier).

***Response: Done***

11. P.23. L.4. "was evident from the IR spectrum". This seems to be the first time we have heard of IR spectral analysis.

***Response: This phrase should not have been included. It has been deleted.***

12. Fig.1. Some readers may not know what the abbreviations 'COSY' (=COrrelated SpectroscopY) and 'HMBC' (= heteronuclear multi-bond correlation) mean

***Response: Written in full.***

13. S1 Please define HRESIMS (= high resolution electrospray ionisation mass spectroscopy)

***Response: Defined in the main text***

14. S4. The text is illegible.

***Response: A higher resolution of S4 Fig has been provided.***

It would be helpful to reviewers if the lines were numbered (consecutively from the beginning).

***Response: Done***

**Methods**

The methods are standard, but are not adequately described.
The main objection is that the material studied (herbal remedies) is not described.

***Response: the identity of the herbal remedies have been included.***

**Results**

Some of the figures are illegible, or barely legible.

***Response: A higher resolution of S4 Fig has been provided.***

**Specific comments to be addressed from Reviewer #2**

1) Table 1, number of people successfully treated is not an appropriate heading since you said earlier that ‘According to the practitioners, their efficacy claims are based on the number of people who experience reduction or relief from symptoms as a result of using their products’. So it should be coined as the number of people relieved of symptoms and not successfully treated. Given that no laboratory tests were done to determine parasite loads, it is subjective to conclude that the patients were treated successfully.

***Response: The column in question has been removed from Table 1.***

2) Statistical analysis for comparisons of different extracts and concentrations for onchocerca and schistosoma will enhance the credibility of findings given that you ran the assays in quadruplicates and triplicates repeatedly.This will reveal if the differences observed are statistically significant in different concentrations or at the IC50. A good example is Table 5.

***Response: The SD values for the onchocerca and schistosoma analyses have been included in relevant tables.***

**Minor Points**

3) We recommend that the quality of traditional medicines be improved (Author summary)

***Response: Corrected.***

4) Primary screening of adult worms and microfilariae, Secondary screening on adult worms

It should be “screening against adult worms” instead of “screen of adult worms”

***Response: Corrected.***

5) Correct from GCMSNMR to GC/MS/NMR in the manuscript as they are different techniques.

***Response: Originally, the techniques were not presented as one acronym (GCMSNMR) but separated by commas as follows: GCMS, NMR, MS.***

***In line with the comment that these analytical techniques are different, and to reflect the way in which they were employed, as captured in the subsequent statements, the correction made is as follows: GC/MS, NMR and HRESIMS. Further, based on the comment form Reviewer 1, the full names have also been provided.***

6) Discussion section, ‘however, is on the rise and possess’ it should be poses

***Response: Corrected.***

7) Introduction section ‘high cost of bringing new drugs to the market’

***Response: Corrected.***

**Results**

1) Cytotoxicity assessment of extracts. The 50% cytotoxic concentrations (CC50) of the cells were determined by microscopic examinations.

Looking at Table 4, the cytotoxicity was done at 200 µg/mL only. So the CC50 was not determined as stated in the methodology. The selectivity index (SI) column should be included if the CC50 was determined. Also, consider including this aspect in your abstract.

***Response: That is a valid point. Only preliminary cytotoxicity screening on LLC-MK2 cells was conducted; hence, the CC50 was not determined as indicated. The necessary correction has been effected in Table 4.***

**Conclusions**

2) Looking at the results you obtained for the antionchocercal activity of extracts, all the active extracts are dichloromethane extracts. Most filaricidal activities of plants tested come from non-polar extracts. This could be backed up in the discussion section with publications from other authors who had similar findings.

***Response: The suggestion has been included in lines 656-657 of the document with track changes and lines 502 and 503 in the document without track changes. The supporting references are 49 and 50.***

**Specific comments from Reviewer #3**

**Results**

The objectives of the study are clearly defined, but without a clear testable hypothesis stated.

***Response: A hypothesis has been included in the penultimate paragraph under the Introduction as follows: TMs that are used locally against NTDs, contain compounds that possess activity against the disease-causing pathogens. See lines 135-136 of the document with track changes and lines 133-134in the document without track changes.***
