# Supplementary material for: Antischistosomal, antionchocercal and antitrypanosomal potentials of some Ghanaian traditional medicines and their constituents
Source: PLoS Negl Trop Dis. 2020 Dec 31;14(12):e0008919. doi: 10.1371/journal.pntd.0008919 (PMC7810346; doi:10.1371/journal.pntd.0008919)
Supplement: Attachment — Submitted filename: Cover letter PNTD-D-20-00655R2_edit.docx [file pntd.0008919.s010.docx]

Department of Chemistry

School of Physical and Mathematical Sciences

College of Basic and Applied Sciences

University of Ghana

Legon.

October 17, 2020

The Editors

PLOS Neglected Tropical Diseases

Dear Editors,

**RE: MODIFICATIONS FOR PNTD-D-20-00655R2**

I write to kindly indicate that all requested modifications for the above manuscript have been responded to. Per the recommendations, both clean and marked-up copies of the manuscripts have been uploaded.

The following changes were made:

1. Copyright or trademark symbols accompanying Phyto-Laria®", "Whatman®", "Alamar Blue®" and "Varioskan™" have all been deleted from the manuscript.

2. Regarding ethical approval for the study, formal consent was obtained verbally. A statement to that effect has been included in the manuscript in lines 154 and 155.

3. Figure 1 has been removed from the manuscript and formatted to meet journal specifications. It has been uploaded as a separate file.

4. The supplementary figures have also been formatted and removed from the manuscript file. They have been uploaded as supporting information and the legend for each one of them is listed in the manuscript.

Kindly note that Emmanuella Bema Twumasi has informed me of her confirmation of authorship on the manuscript.

Please, do not hesitate to notify me of any outstanding issue.

Yours faithfully,

Professor Dorcas Osei-Safo (PhD)

Email: [dosei-safo@ug.edu.gh](mailto:dosei-safo@ug.edu.gh)

Mob: +23320816937
